# Enhanced Stability of MAPbI_3_ Perovskite Solar Cells using Poly(p-chloro-xylylene) Encapsulation

**DOI:** 10.1038/s41598-019-51945-9

**Published:** 2019-10-29

**Authors:** Hyojung Kim, Jiyong Lee, Bora Kim, Hye Ryung Byun, Sung Hyuk Kim, Hye Min Oh, Seunghyun Baik, Mun Seok Jeong

**Affiliations:** 1 Department of Energy Science, Sungkyunkwan University, Suwon, 16419 Republic of Korea; 2 Department of Physics, Sogang University, Seoul, 04107 Republic of Korea; 3 Center for Integrated Nanostructure Physics, Institute for Basic Science (IBS), Suwon, 16419 Republic of Korea; 4 School of Mechanical Engineering, Sungkyunkwan University, Suwon, 16419 Republic of Korea

**Keywords:** Materials science, Optics and photonics

## Abstract

We demonstrated an effective poly(p-chloro-xylylene) (Parylene-C) encapsulation method for MAPbI_3_ solar cells. By structural and optical analysis, we confirmed that Parylene-C efficiently slowed the decomposition reaction in MAPbI_3_. From a water permeability test with different encapsulating materials, we found that Parylene-C-coated MAPbI_3_ perovskite was successfully passivated from reaction with water, owing to the hydrophobic behavior of Parylene-C. As a result, the Parylene-C-coated MAPbI_3_ solar cells showed better device stability than uncoated cells, virtually maintaining the initial power conversion efficiency value (15.5 ± 0.3%) for 196 h.

## Introduction

Hybrid organo-lead halide perovskites have received significant attention for use as absorber materials in solar cells, because of their high absorption coefficient^1^, tunable band gap^2,3^, high carrier mobility^4^, and simple solution-processing synthesis method^5,6^. Since Kojima *et al*. reported methylammonium lead iodide (CH_3_NH_3_PbI_3_)-based perovskite solar cells (PSCs) in 2009^7^, various synthesis methods and device architectures have been studied^8–11^, and the highest power conversion efficiency (PCE), 24.2%, was recently achieved using a double-layered halide structure^12,13^. However, the inherent instability of CH_3_NH_3_PbI_3_ remains a major concern in perovskite research^14–16^. For example, Conings *et al*. reported a significant structural change with a 75% drop in the PCE due to degraded CH_3_NH_3_PbI_3_ in ambient conditions^17^, and Leijtens *et al*. also observed a 50% decay of the initial performance of unpassivated PSCs within 5 h^18^. Several attempts have been made to improve the stability of PSCs by modifying the crystal structure^19^ or replacing the hole-transporting layer (HTL) with carbon nanotube–polymer composites^20^. More recently, Cheacharoen *et al*. reported that ethylene vinyl acetate encapsulation retained 90% of the initial device performance after 200 temperature cycles between −40 °C and 85 °C^21^. However, despite the use of complex chemical compositions^22^ or various passivation layers^21,23,24^, ~10–20% drops in PCE were still observed, and the current research interests have focused more on the thermal stability of perovskite. Therefore, to overcome the remaining concerns, it is necessary to find an alternative encapsulating material.

In this work, we suggest an effective poly(p-chloro-xylylene) (Parylene-C) encapsulation method for CH_3_NH_3_PbI_3_ (MAPbI_3_) solar cells. The passivation was verified by monitoring the time-dependent optical properties of a MAPbI_3_ perovskite film, including the UV–Vis absorption and photoluminescence (PL) spectra. As a result, Parylene-C successfully passivated a MAPbI_3_ perovskite film from reaction with water, owing to its hydrophobic behavior. The photovoltaic performance of Parylene-C-coated PSCs was investigated at various air-exposure times, and the Parylene-C-coated PSCs showed improved stability, maintaining almost the initial PCE values (15.5 ± 0.3%) for 196 h.

## Results and Discussion

**Figure 1:**
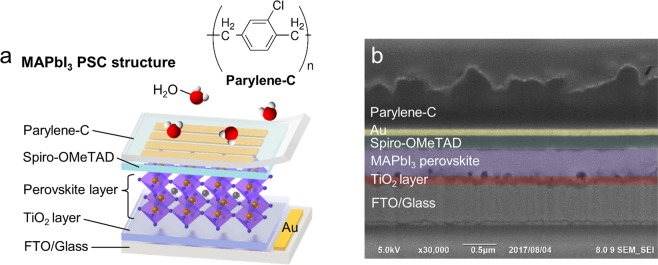
Structure of the Parylene-C-coated MAPbI_3_ PSC. (**a**) Schematic of the MAPbI_3_ PSC structure with Parylene-C encapsulation and the chemical structure of Parylene-C. (**b**) Cross-sectional SEM image of the Parylene-C-coated MAPbI_3_ PSC.

The device configuration of the Parylene-C-coated MAPbI_3_ solar cell is shown in Fig. 1a. MAPbI_3_ perovskite was fabricated using the two-step spin-casting method^25^, with the TiO_2_ and Spiro-OMeTAD used for the electron and hole transporting materials, respectively. The 100-nm-thick gold electrodes and Parylene-C polymer were sequentially deposited on the top of the MAPbI_3_ solar cells, and the active solar cell area was 0.15 cm^2^ (see Supplementary Information for details). The chemical structure of Parylene-C - phenyl rings, with one chlorine and two methylene groups, is shown at the top of Fig. 1a. We confirmed the chemical structure of Parylene-C via Fourier transform infrared (FTIR) spectroscopy (Fig. S1). Parylene-C showed the absorption peaks at 1045 cm^−1^ and 1492 cm^−1^, corresponding to the vibration energy of chlorine and phenyl groups, respectively^26^. The two C-H stretching modes related to methyl groups were also observed at 2857 cm^−1^ and 2927 cm^−1^ ^27^. Figure 1b shows a cross-sectional scanning electron microscopy (SEM) image of the Parylene-C-coated MAPbI_3_ solar cell, and we confirmed that the thickness of the Parylene-C coating was about 700 nm. The rough surface of the Parylene-C layer was generated by the focused ion beam milling process (Fig. S2). Generally, Parylene-C shows a low surface roughness value of 4.1 ± 0.4 nm because of using a vapor-deposition technique^28^.

**Figure 2:**
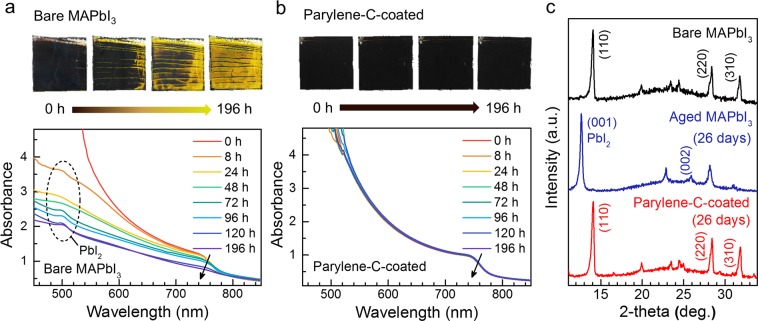
Parylene-C passivation effect in ambient conditions. Time-series photographs and linear absorption spectra of (**a**) bare MAPbI_3_ and (**b**) Parylene-C-coated MAPbI_3_. (**c**) XRD spectra of as-prepared bare MAPbI_3_ (black), aged bare MAPbI_3_ (blue), and Parylene-C-coated MAPbI_3_ (red) after 26 d.

First, we measured the time-dependent absorption of bare and Parylene-C-coated MAPbI_3_ in order to trace the decomposition reaction in ambient conditions; we also took photographs simultaneously (Fig. S3). The perovskite films were exposed to a room temperature (26.1 °C ± 2 °C) environment with 40–50% relative humidity for 196 h. As shown in Fig. 2a, we observed that the bare MAPbI_3_ changed from dark brown to yellow, and the absorption peak at 762 nm gradually decreased. We also found that a new absorption peak at 510 nm became dominant, as the entire film of bare MAPbI_3_ became yellow (dashed circle in Fig. 2a). In contrast, the Parylene-C-coated MAPbI_3_ film exhibited no color changes, and the absorption also remained unchanged after 196 h in air (Fig. 2b).

To understand these absorption changes, X-ray diffraction (XRD) analysis of the samples exposed to air for 26 d was performed (Fig. 2c). The predominant XRD peaks observed in the as-prepared bare MAPbI_3_ (black, top) significantly decreased in the aged bare MAPbI_3_ (blue, middle) and a new peak appeared at 12.64°. According to a previous report, this peak corresponds to the (001) diffraction of PbI_2_, which is a well-known by-product of the degradation of MAPbI_3_ perovskite^29^. We confirmed that the absorption change below 550 nm (2.25 eV) in Fig. 2a was due to the formation of PbI_2_ because the band gap energy of PbI_2_ is about 2.30 eV. In contrast, the diffraction patterns of the Parylene-C-coated MAPbI_3_ film were almost identical to those of the as-prepared MAPbI_3_, even after 26 d (red, bottom). This result indicates that the Parylene-C passivation layer effectively slowed the decomposition reaction in the MAPbI_3_ perovskite.

**Figure 3:**
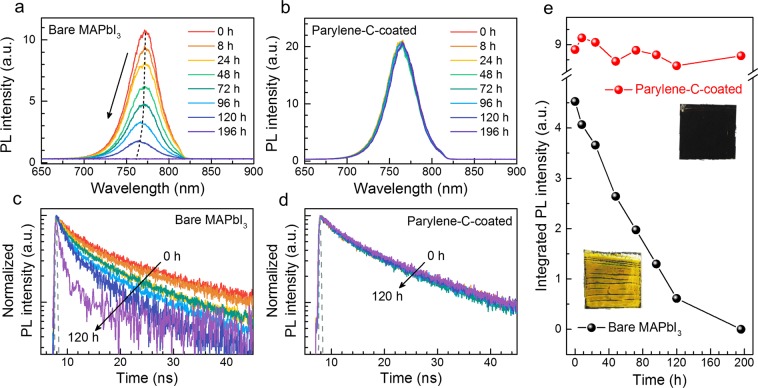
Optical properties of MAPbI_3_ with and without Parylene-C deposition. Time-dependent PL spectra of (**a**) bare and (**b**) Parylene-C-coated MAPbI_3_. TRPL decay profiles of (**c**) bare and (**d**) Parylene-C-coated MAPbI_3_ with the instrument response function (dashed gray line). (**e**) Integrated PL intensity as a function of air-exposure time for bare (black) and Parylene-C-coated MAPbI_3_ (red) (inset: photographs of each sample after 196 h).

We further confirmed the passivation effect of Parylene-C using time-dependent PL as a function of air-exposure time. In Fig. 3a, the PL intensity of the bare MAPbI_3_ decreased dramatically over time, and the PL peak position was slightly blue-shifted (black dashed line). According to a previous report, the blue-shifted PL peak of aged MAPbI_3_ was related to PbI_2_ formation^17^, and we observed an increased PL intensity at 510 nm from the aged MAPbI_3_ (Fig. S4), which is consistent with the band gap of PbI_2_. In contrast, Parylene-C-coated MAPbI_3_ maintained its initial PL intensity and peak position for 196 h (Fig. 3b), showing the effective passivation effect of Parylene-C on MAPbI_3_.

Time-resolved photoluminescence (TRPL) measurements were conducted simultaneously on both bare and Parylene-C-coated MAPbI_3_ in Fig. 3c,d. The integrated PL intensity of the bare MAPbI_3_ was zero after 120 h; thus, we compared the TRPL spectra taken for 120 h (excluding data taken after 196 h). As shown in Fig. 3c, the PL decay curves of the bare MAPbI_3_ decreased gradually with increasing exposure time, whereas those of the Parylene-C-coated MAPbI_3_ remained unchanged for 120 h (Fig. 3d). We calculated the average lifetime from two decay components fitted to a bi-exponential function and plotted the changes as a function of air-exposure time (Fig. S5). The carrier lifetime in the bare MAPbI_3_ decreased remarkably from the initial value of 8.67 ns to 0.99 ns because of the decomposition reaction. In contrast, Parylene-C-coated MAPbI_3_ initially exhibited a longer carrier lifetime than the bare film, i.e., 9.19 ns, maintained for 120 h.

**Figure 4:**
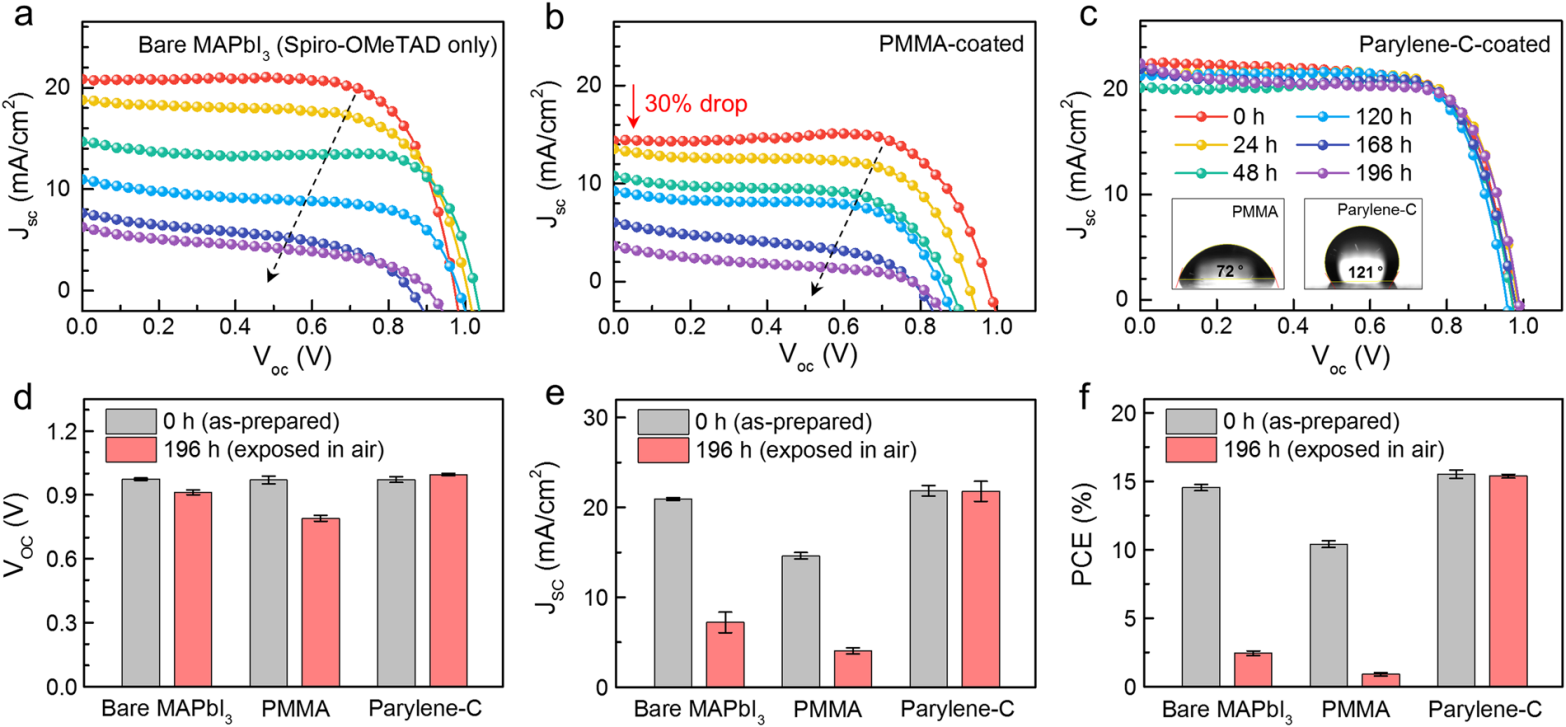
Time-dependent photovoltaic characteristics depending on PSC encapsulation. Time-dependent *J*-*V* curves of the (**a**) bare, (**b**) PMMA-coated, and (**c**) Parylene-C-coated MAPbI_3_ PSCs for 196 h (inset: images of a water drop on PMMA and Parylene-C showing contact angles of 72° and 121°, respectively). Comparison of (**d**) *V*oc, (**e**) *J*sc, and (**f**) PCE of MAPbI_3_ solar cells with different encapsulating layers before (gray) and after (red) exposure for 196 h.

Finally, we investigated the photovoltaic performance of Parylene-C-coated MAPbI_3_ solar cells in terms of the effect of Parylene-C encapsulation. The solar cell performance was measured under the AM 1.5 G illumination with a power density of 100 mW cm^−2^ (see Supplementary Information for details). In Fig. S6, the comparison of current density–voltage (*J*–*V*) curves before and after Parylene-C deposition revealed that the MAPbI_3_ PSC was not damaged during the vapor-deposition process. Figure 4 shows time-dependent photovoltaic characteristics for the bare, polymethyl methacrylate (PMMA)-coated, and Parylene-C-coated MAPbI_3_ PSCs. We selected PMMA for comparison, as it is the most commonly used polymer for PSC encapsulation. Interestingly, the PMMA-coated PSCs showed a 30% drop from their initial efficiency (Fig. 4b) because chlorobenzene, a pre-dissolution of PMMA and Spiro-OMeTAD, can damage Spiro-OMeTAD during PMMA coating^30^. This result indicates that the use of PMMA limits the selection of hole-transport materials in solar cells, although it is among the most widely used of various passivation polymers. On the contrary, Parylene-C can be used in combination with various hole-transport materials without affecting the initial device performance. In terms of device stability, the solar cell performance of the bare and PMMA-coated PSCs decreased dramatically after exposure to ambient conditions, and these cells were completely degraded within 196 h (Fig. 4a,b). In contrast, the PCE of the Parylene-C-coated PSC exhibited no notable change over a period of 196 h (Fig. 4c).

To explain how Parylene-C can efficiently retain the initial performance of MAPbI_3_ PSC, we further performed FTIR spectroscopy. As shown in Fig. S7, two N–H stretching modes around 3150 cm^−1^ were remarkably suppressed for aged MAPbI_3_, and a new absorption peak appeared at 3466 cm^−1^, corresponding to the vibration energy of hydroxyl groups (−OH). This finding agrees well with a previous report^31^ on hydrated compounds in aged MAPbI_3_ resulting from adsorption of water vapor. Note that humid conditions are the main cause of degradation in MAPbI_3_ perovskite, and therefore, to improve stability of the PSC, it is necessary to prevent water penetration. Fortunately, Parylene-C has been known to have low water permeability of 0.14 cm^3^ ·mil/(100 in^2^ 24 hr ·atm) at 23 °C^32^, which correlated well with our contact angle data (the inset of Fig. 4c). The surface of PMMA showed a contact angle of 72°, which were in the hydrophilic range (<90°)^33^. In contrast, Parylene-C showed hydrophobic behavior, with a considerably higher contact angle of 121°.

We tested the water permeability of these encapsulating materials (Video S1). We prepared Spiro-OMeTAD- and PMMA-coated MAPbI_3_ as well as bare and Parylene-C-coated MAPbI_3_. The bare and Spiro-OMeTAD-coated MAPbI_3_ perovskite films turned yellow on exposure to water drops, revealing that the hole transporting material could not protect against reaction with water. Although the PMMA-coated MAPbI_3_ initially remained unchanged, the decomposition reaction occurred within 6 min. Yoo *et al*. reported that a part of PMMA molecular chain is hydrophilic; thus, water molecules can hydrolyze the ester groups in PMMA and break down the PMMA structure^30^. This is consistent with our results, indicating that PMMA is unable to protect perovskite from water damage. Unlike the other tested materials, Parylene-C effectively passivated the perovskite film against water droplet exposure for 30 min. These results indicate that Parylene-C was able to slow down the degradation of PSC because it could effectively passivate MAPbI_3_ PSC from reaction with water.

For further analysis, we compared the photovoltaic parameters of the as-prepared (0 h) and aged (after 196 h) PSCs with different encapsulating layers. In Fig. 4e,f, the bare and PMMA-coated PSCs showed remarkable decreases in *J*sc and PCE values after 196 h. Interestingly, the *V*oc values hardly changed after 196 h for both the bare and PMMA-coated solar cells (Fig. 4d). Conings *et al*. reported similar small decreases of *V*oc, from aged MAPbI_3_ PSCs, and revealed that *J*sc was influenced more by degradation because perovskite degradation increased resistance and recombination near the interface between the perovskite and carrier transporting layers^17^. On the basis, we considered that the huge decrease of *J*sc in our data was also attributable to perovskite layer degradation. Lastly, the Parylene-C-coated PSC showed no noticeable change to any of the parameters; therefore, we confirmed that Parylene-C could effectively encapsulate the MAPbI_3_ solar cells for 196 h.

## Conclusions

We demonstrated successful encapsulation of MAPbI_3_ solar cells by Parylene-C deposition. By structural and optical analyses, we systemically investigated the origin of the decomposition reaction in MAPbI_3_ and confirmed that Parylene-C can efficiently slow down the decomposition reaction in the MAPbI_3_ films. In particular, Parylene-C can efficiently isolate MAPbI_3_ perovskite from reaction with water, owing to its hydrophobic character, and as a result, the Parylene-C-coated MAPbI_3_ solar cells maintained almost the initial PCE values (15.5 ± 0.3%) for 196 h. On the basis of this work, we believe that the (p-xylylene) type polymers have shown the potential to improve the lifetime of organo-lead halide perovskite in future photovoltaic applications.

## Supplementary information

Supplementary Information

Supplementary Video

## References

[1] Green MA, Ho-Baillie A, Snaith HJ (2014). The Emergence of Perovskite Solar Cells. Nat. Photon..

[2] Sutton RJ (2016). Bandgap-Tunable Cesium Lead Halide Perovskites with High Thermal Stability for Efficient Solar Cells. Adv. Energy Mater..

[3] Eperon GE (2014). Formamidinium Lead Trihalide: a Broadly Tunable Perovskite for Efficient Planar Heterojunction Solar Cells. Energy Environ. Sci..

[4] Wehrenfennig C, Eperon GE, Johnston MB, Snaith HJ, Herz LM (2014). High Charge Carrier Mobilities and Lifetimes in Organolead Trihalide Perovskites. Adv. Mater..

[5] Lee MM, Teuscher J, Miyasaka T, Murakami TN, Snaith HJ (2012). Efficient Hybrid Solar Cells Based on Meso-Superstructured Organometal Halide Perovskites. Science.

[6] Nie W (2015). High-Efficiency Solution-Processed Perovskite Solar Cells with Millimeter-Scale Grains. Science.

[7] Kojima A, Teshima K, Shirai Y, Miyasaka T (2009). Organometal Halide Perovskites as Visible-Light Sensitizers for Photovoltaic Cells. J. Am. Chem. Soc..

[8] Burschka J (2013). Sequential Deposition as a Route to High-Performance Perovskite-Sensitized Solar Cells. Nature.

[9] Zhou H (2014). Interface Engineering of Highly Efficient Perovskite Solar Cells. Science.

[10] Sutherland BR (2015). Perovskite Thin Films via Atomic Layer Deposition. Adv. Mater..

[11] Chen C-W (2014). Efficient and Uniform Planar-Type Perovskite Solar Cells by Simple Sequential Vacuum Deposition. Adv. Mater..

[12] NREL. Best Research-Cell Efficiencies. *National Renewable Energy Laboratory*, https://www.nrel.gov/pv/assets/images/thumb-best-research-cell-efficiencies-190416.png (2019).

[13] Jung EH (2019). Efficient, Stable and Scalable Perovskite Solar Cells using Poly(3-hexylthiophene). Nature.

[14] Han Y (2015). Degradation Observations of Encapsulated Planar CH_3_NH_3_PbI_3_ Perovskite Solar Cells at High Temperatures and Humidity. J. Mater. Chem. A.

[15] Niu G (2014). Study on the Stability of CH_3_NH_3_PbI_3_ Films and the Effect of Post-Modification by Aluminum Oxide in All-Solid-State Hybrid Solar Cells. J. Mater. Chem. A.

[16] Noel NK (2014). Enhanced Photoluminescence and Solar Cell Performance via Lewis Base Passivation of Organic–Inorganic Lead Halide Perovskites. ACS Nano.

[17] Conings B (2015). Intrinsic Thermal Instability of Methylammonium Lead Trihalide Perovskite. Adv. Energy Mater..

[18] Leijtens T (2013). Overcoming Ultraviolet Light Instability of Sensitized TiO_2_ with Meso-Superstructured Organometal Tri-halide Perovskite Solar Cells. Nat. Commun..

[19] Noh JH, Im SH, Heo JH, Mandal TN, Seok SI (2013). Chemical Management for Colorful, Efficient, and Stable Inorganic–Organic Hybrid Nanostructured Solar Cells. Nano Lett..

[20] Habisreutinger SN (2014). Carbon Nanotube/Polymer Composites as a Highly Stable Hole Collection Layer in Perovskite Solar Cells. Nano Lett..

[21] Cheacharoen R (2018). Design and Understanding of Encapsulated Perovskite Solar Cells to Withstand Temperature Cycling. Energy Environ. Sci..

[22] Niu G, Li W, Li J, Liang X, Wang L (2017). Enhancement of Thermal Stability for Perovskite Solar Cells Through Cesium Doping. RSC Adv..

[23] Rizzo, A. *et al*. Effects of Thermal Stress on Hybrid Perovskite Solar Cells with Different Encapsulation Techniques. *IEEE International Reliability Physics Symposium (IRPS)*, 2–6 (2017).

[24] Park DY, Byun HR, Kim H, Kim B, Jeong MS (2018). Enhanced Stability of Perovskite Solar Cells using Organosilane-treated Double Polymer Passivation Layers. J. Korean Phys. Soc..

[25] Lee J, Menamparambath MM, Hwang J-Y, Baik S (2015). Hierarchically Structured Hole Transport Layers of Spiro-OMeTAD and Multiwalled Carbon Nanotubes for Perovskite Solar Cells. Chem. Sus. Chem..

[26] Callahan RRA, Puden KG, Raupp GB, Beaudoin SP (2003). Downstream Oxygen Etching Characteristics of Polymers from the Paryelene Family. J. Vac. Sci. Technol. B.

[27] Kim HT, Koo T, Park C (2010). Parylene-C Thin Films Deposited on Polymer Substrates Using a Modified Chemical Vapor Condensation Method. Korean J. Chem. Eng..

[28] Jean J, Wang A, Bulović V (2016). *In Situ* Vapor-Deposited Parylene Substrates for Ultra-Thin, Lightweight Organic Solar Cells. Org. Electron..

[29] Wang C (2016). Degradation of Co-Evaporated Perovskite Thin Film in Air. Chem. Phys. Lett..

[30] Yoo JS (2017). Dual Function of a High-Contrast Hydrophobic-Hydrophilic Coating for Enhanced Stability of Perovskite Solar Cells in Extremely Humid Environments. Nano Res..

[31] Yang J, Siempelkamp BD, Liu D, Kelly TL (2015). Investigation of CH_3_NH_3_PbI_3_ Degradation Rates and Mechanisms in Controlled Humidity Environments Using *in Situ* Techniques. ACS Nano.

[32] Kim H (2018). Polymer Passivation Effect on Methylammonium Lead Halide Perovskite Photodetectors. J. Korean Phys. Soc..

[33] Nuraje N, Khan WS, Lei Y, Ceylan M, Asmatulu R (2013). Superhydrophobic Electrospun Nanofibers. J. Mater. Chem. A.

