# Supplementary material for: Enhanced Stability of MAPbI3 Perovskite Solar Cells using Poly(p-chloro-xylylene) Encapsulation
Source: Sci Rep. 2019 Oct 29;9:15461. doi: 10.1038/s41598-019-51945-9 (PMC6820723; doi:10.1038/s41598-019-51945-9)
Supplement: Supplementary file 1 — Supplementary Information [file 41598_2019_51945_MOESM1_ESM.docx]

**Supplementary Information**

**Enhanced Stability of MAPbI_3_ Perovskite Solar Cells using Poly(p-chloro-xylylene) Encapsulation**

Hyojung Kim,^1^ Jiyong Lee,^1^ Bora Kim,^1^ Hye Ryung Byun,^2^ Sung Hyuk Kim,^1^ Hye Min Oh,^1^ Seunghyun Baik,^3,4^ and Mun Seok Jeong^1,3^

^1^ Department of Energy Science, Sungkyunkwan University, Suwon, 16419, Republic of Korea.

^2^ Department of Physics, Sogang University, Seoul, 04107, Republic of Korea.

^3^ Center for Integrated Nanostructure Physics, Institute for Basic Science (IBS), Suwon, 16419, Republic of Korea.

^4^ School of Mechanical Engineering, Sungkyunkwan University, Suwon 16419, Republic of Korea.

**Experimental Section**

***Chemicals and synthesis of MAPbI_3_ films:*** CH_3_NH_3_PbI and PbI_2_ (≥99.99%) were purchased from Dyesol Limited (Australia) and Xi’an Polymer Light Technology Corp. (China), respectively. *N*-methyl-2-pyrrolidone (NMP) and diethyl ether (DEE) (anhydrous, 99.8%) were purchased from Sigma Aldrich and Alfa Aesar, respectively. CH_3_NH_3_PbI and PbI_2_ were stirred in a mixture of NMP at 70 °C for 12 h to obtain a 42 wt% precursor solution. The precursor was spin-coated onto a glass substrate at 4500 rpm for 15 s, and the solution-coated substrate was vertically dipped in a DEE bath (about 100 mL) immediately. A dark brown MAPbI_3_ film formed in 2 min, and the substrate was removed from the bath and dried using a nitrogen gun.

***Characterization:*** The cross-sectional scanning electron microscopy (SEM) image was obtained using a focused ion beam scanning electron microscope (FIB-SEM, JIB-4601F, JEOL). The UV-Vis absorption and FTIR spectra were measured using V-670 (JASCO) and FT/IR-4700 (JASCO) spectrometers, respectively. X-ray diffraction (XRD) patterns were obtained by using an XRD diffractometer (D8 ADVANCE, Bruker). Photoluminescence (PL) and time-resolved PL (TRPL) spectroscopy were performed using a confocal microscope system (NTEGRA SPECTRA, NT-MDT) with a 100× objective lens (NA 0.7) and a 150 lines/mm grating with a 400 nm blaze. For the PL measurement, a 405 nm solid-state laser (0.2 μW) was used as the excitation source, and spectra were collected using a thermoelectrically cooled CCD detector (exposure time: 0.1 s). For the TRPL measurement, a 405-nm pulsed laser with a repetition rate of 20 MHz was used with the same excitation power of 0.2 μW. TRPL decay curves were collected with 10 s integration time, and a high-speed photomultiplier tube detector (PMC-100, Photonic Solutions) was used for the time-correlated single photon counting system.

***Fabrication and characterization of MAPbI_3_ solar cells:*** The architecture of the MAPbI_3_ perovskite solar cell was FTO/TiO_2_/MAPbI_3_/spiro-OMeTAD/Au.^[1]^ The TiO_2_ layer was deposited on an FTO-coated glass substrate (Pilkington, TEC 8) using chemical bath deposition (0.2 M TiCl_4_ aqueous solution in the oven at 60°C for 1 h). The TiO_2_-deposited FTO/glass substrate was cleaned using deionized (DI) water and dried on a hot plate at 100°C for 1 h. For the MAPbI_3_ perovskite layer, CH_3_NH_3_I and PbI_2_ (molar ratio of 1:1) were mixed in γ-butyrolactone and dimethyl sulfoxide (7:3 *v/v*) at 70°C for 12 h. The solution was spin-coated onto the TiO_2_/FTO substrate by spin-coating the MAPbI_3_ solution at 1000 rpm for 30 s, and then drop-casting toluene at 5000 rpm for 20 s. The MAPbI_3_/TiO_2_/FTO substrate was annealed at 100°C for 30 min under ambient conditions. Next, 72.3 mg mL^−1^ of spiro-OMeTAD in chlorobenzene was mixed with 28.8 µL of 4-tert-butylpyridine and 17.5 µL of Li-TFSI (520 mg mL^−1^ Li-TFSI in acetonitrile) solution and spin-coated at 4000 rpm for 1 min. After that, the spiro-OMeTAD/MAPbI_3_/TiO_2_/FTO substrate was dried under vacuum at room temperature for 2 h. The MAPbI_3_ and spiro-OMeTAD films were deposited in a nitrogen-filled glove box. Then gold electrodes about 100 nm thick were thermally deposited, after the solar cells were dried in vacuum for 12 h.^[1]^ The active solar cell area was maintained constant (0.15 cm^2^) using a photomask. For the Parylene-C encapsulation, a Parylene coater (NPCR-400A, Nuricell) was operated with the following steps: vaporization (170°C), pyrolysis (690°C), and polymerization (20°C). Parylene-C was formed with a deposition rate of 1 µm/h under 8.0 mTorr, and 1.68 g of the Parylene-C precursor was used for the 700-nm-thick Parylene-C deposition. Contact angle measurement was performed using a contact angle analyzer (SEO Phoenix 300). The *J*–*V* characteristics were measured in the reverse scan direction, using a digital source meter (Keithley 2400), under the AM 1.5 G illumination with a power density of 100 mW cm^−2^ (Oriel, Sol3ATM).

**
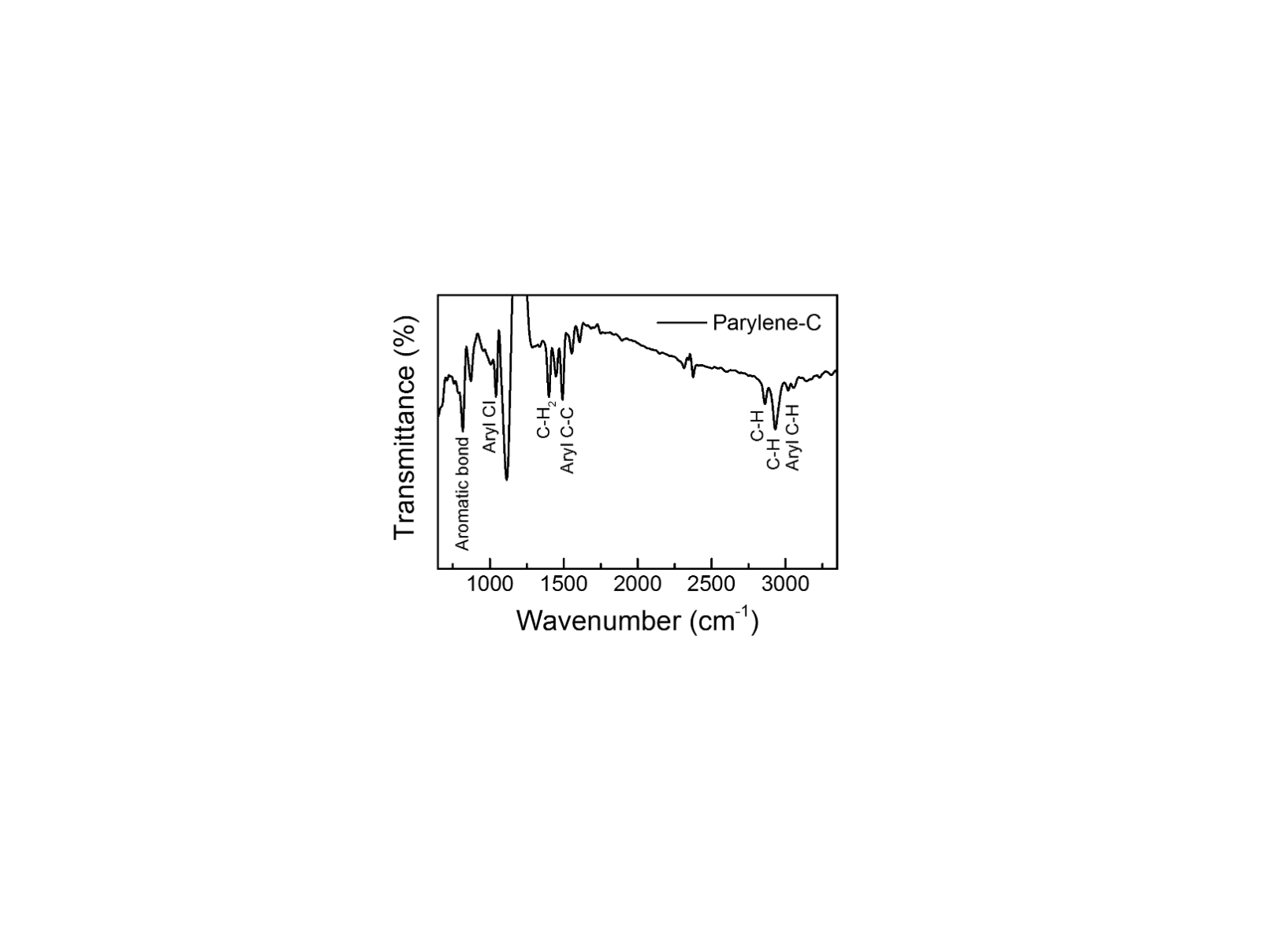
**

**Figure S1.** FTIR spectrum of Parylene-C polymer.

**
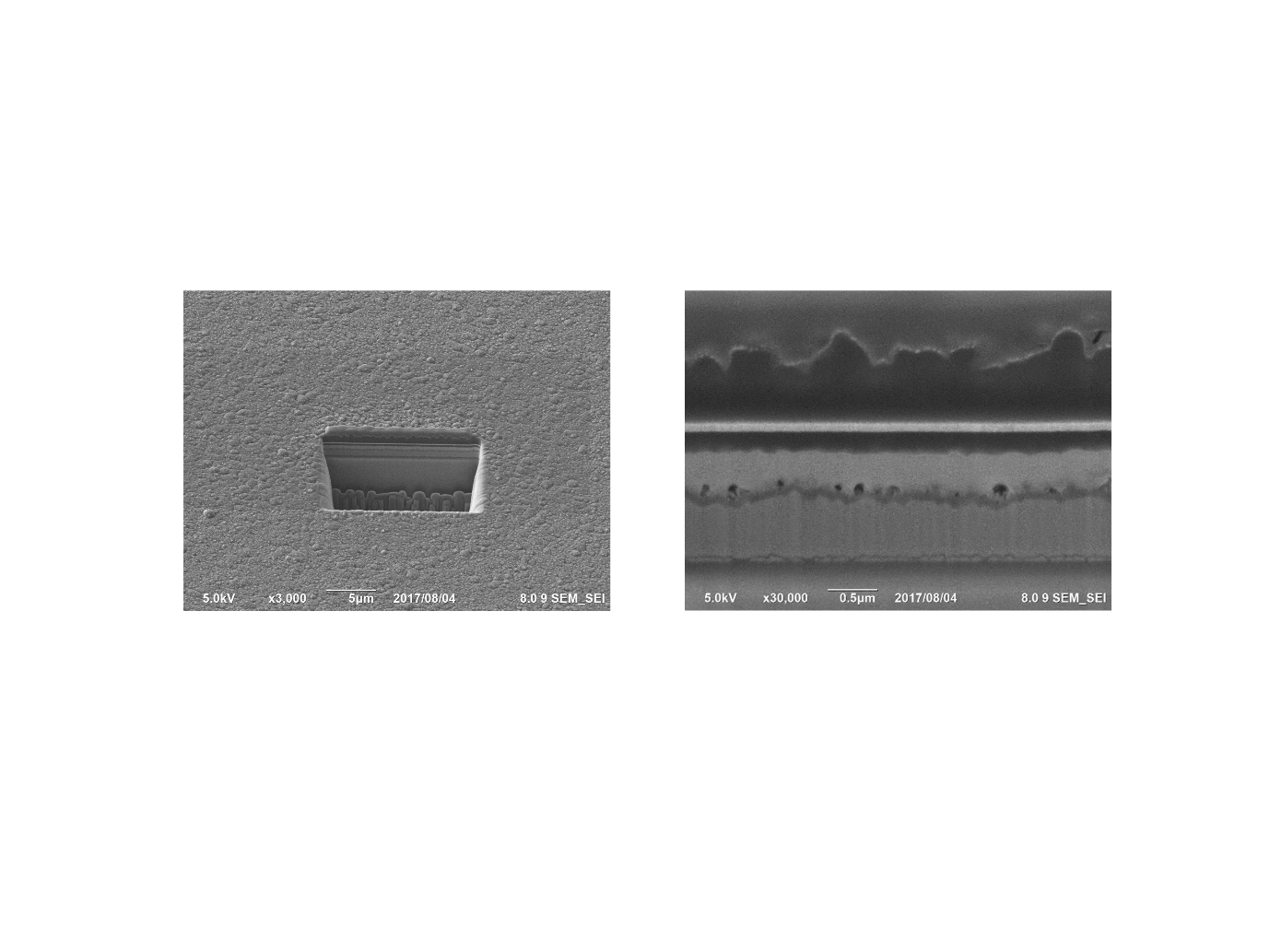
**

**Figure S2.** FIB-SEM image of the Parylene-C-coated MAPbI_3_ solar cell.

**
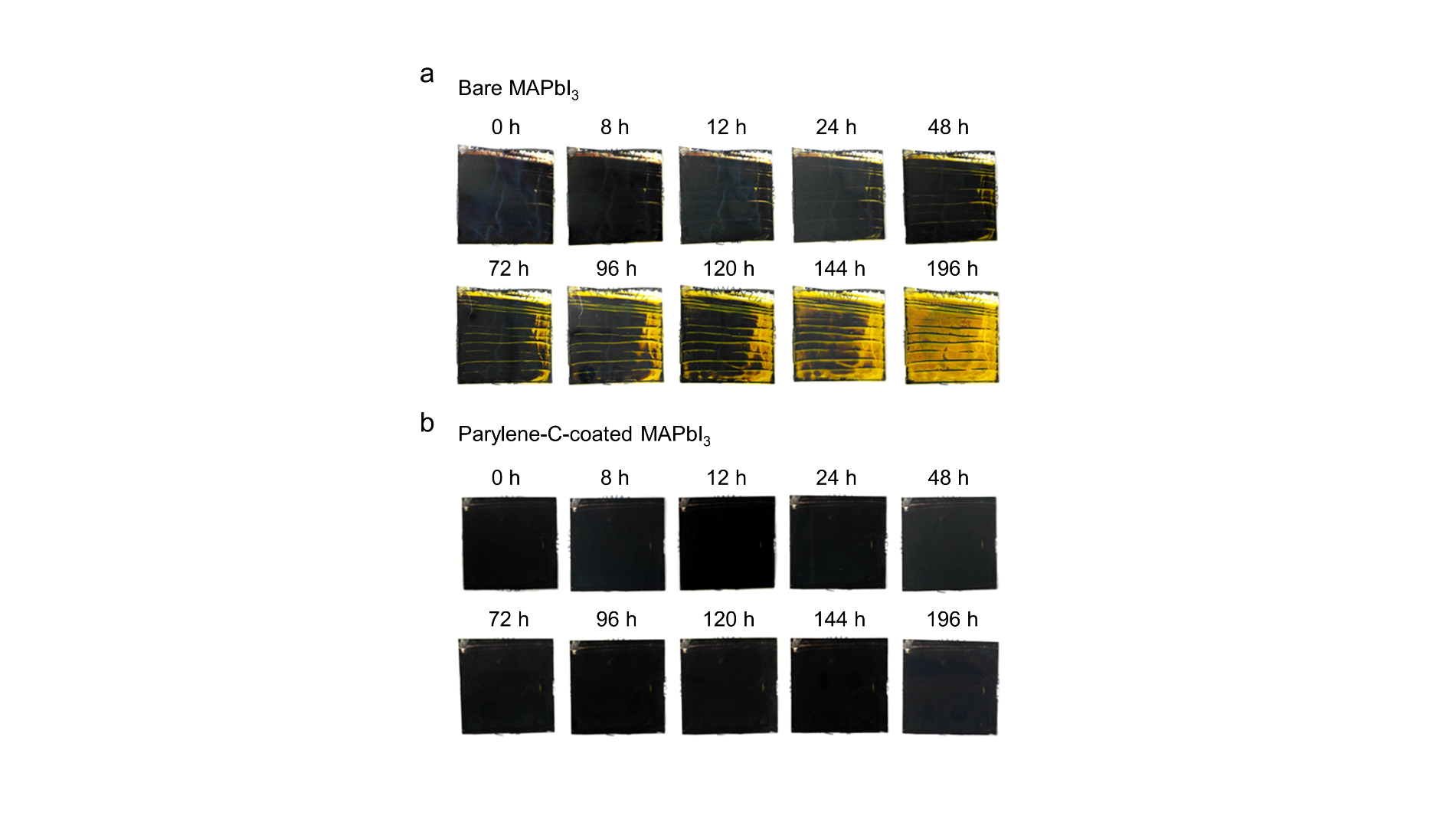
**

**Figure S3.** Time-series photographs of the (a) bare and (b) Parylene-C-coated MAPbI_3_.

**
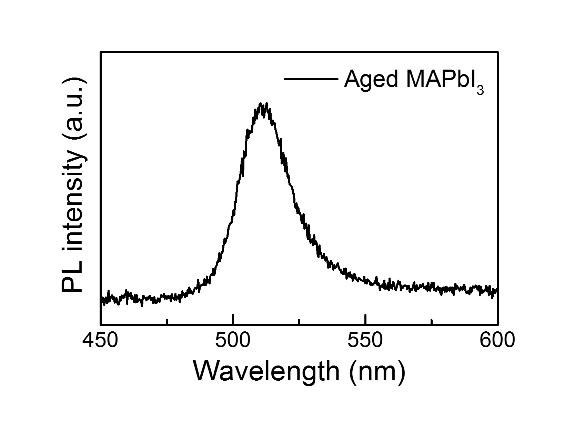
**

**Figure S4.** PL spectrum of the aged MAPbI_3_ film. The PL peak was observed around 510 nm, which is consistent with the band gap of PbI_2_.


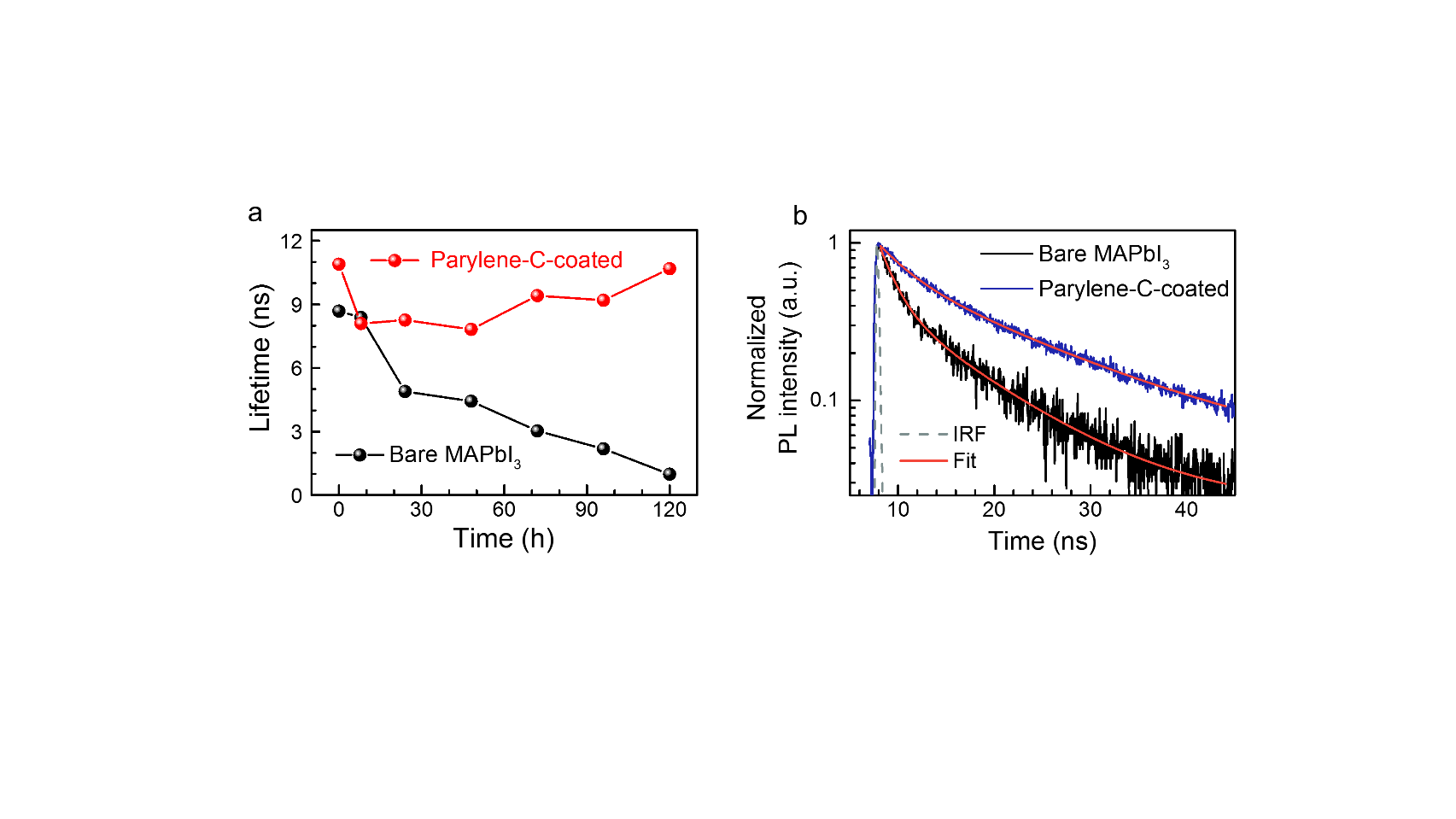


**Figure S5.** (a) Average carrier lifetimes of the bare (black) and Parylene-C-coated (red) MAPbI_3_ as a function of air-exposure time. (b) TRPL decay profiles of the bare (black) and Parylene-C-coated (blue) MAPbI_3_ after 96 h.

To calculate the average carrier lifetime, we fit the TRPL decay profile (red line) to a bi-exponential function given by

$\text{y}\text{ }\text{=}\text{ }\text{y}_{\text{0}}\text{+}{\text{ }\text{a}}_{\text{1}}\text{*exp(-x/}\text{t}_{\text{1}}\text{)}\text{ }\text{+}{\text{ }\text{a}}_{\text{2}}\text{*exp(-x/}\text{t}_{\text{2}}\text{)}$, (1)

where *y*_0_ is an offset, *a_1_* and *a_2_* are weight constants, and *t*_1_ and *t*_2_ are time decay constants.

From these fitting components, we calculated the average amplitudes *A*_1_ and *A*_2_ and the average carrier lifetime as follows:

$$\text{A}_{\text{1}}\left( \text{\%} \right)\text{=}\frac{\text{a}_{\text{1}}}{\text{a}_{\text{1}}\text{+}\text{a}_{\text{2}}}\text{×100 }$$

(2)

$$\text{A}_{\text{2}}\left( \text{\%} \right)\text{=}\frac{\text{a}_{\text{2}}}{\text{a}_{\text{1}}\text{+}\text{a}_{\text{2}}}\text{×100}$$

$$\text{Average carrier lifetime (ns)=}\frac{\text{A}_{\text{1}}{t_{1}}^{2}\text{+}\text{ }\text{A}_{\text{2}}{t_{2}}^{2}}{\text{A}_{\text{1}}\text{t}_{\text{1}}\text{+}\text{A}_{\text{2}}\text{t}_{\text{2}}}$$

~~
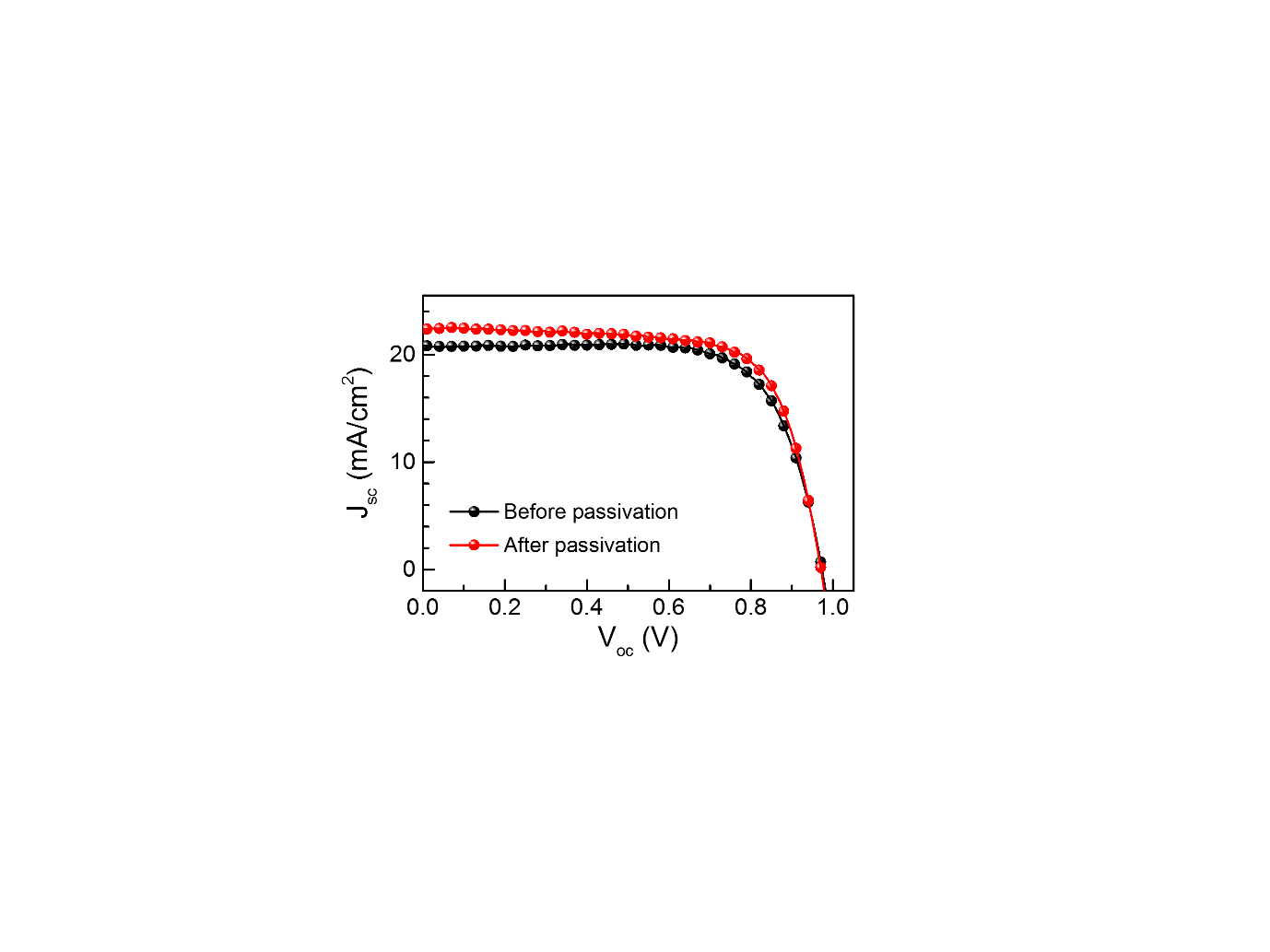
~~

**Figure S6.** *J-V* curves of MAPbI_3_ solar cells before (black) and after (red) Parylene-C deposition.

|  | V_OC_ (V) | J_SC_ (mAcm^-2^) | FF | PCE (%) |
| --- | --- | --- | --- | --- |
| Before encapsulation | 0.97 | 20.9 | 0.72 | 14.5 ± 0.2 |
| After encapsulation | 0.97 | 22.4 | 0.72 | 15.5 ± 0.3 |

**
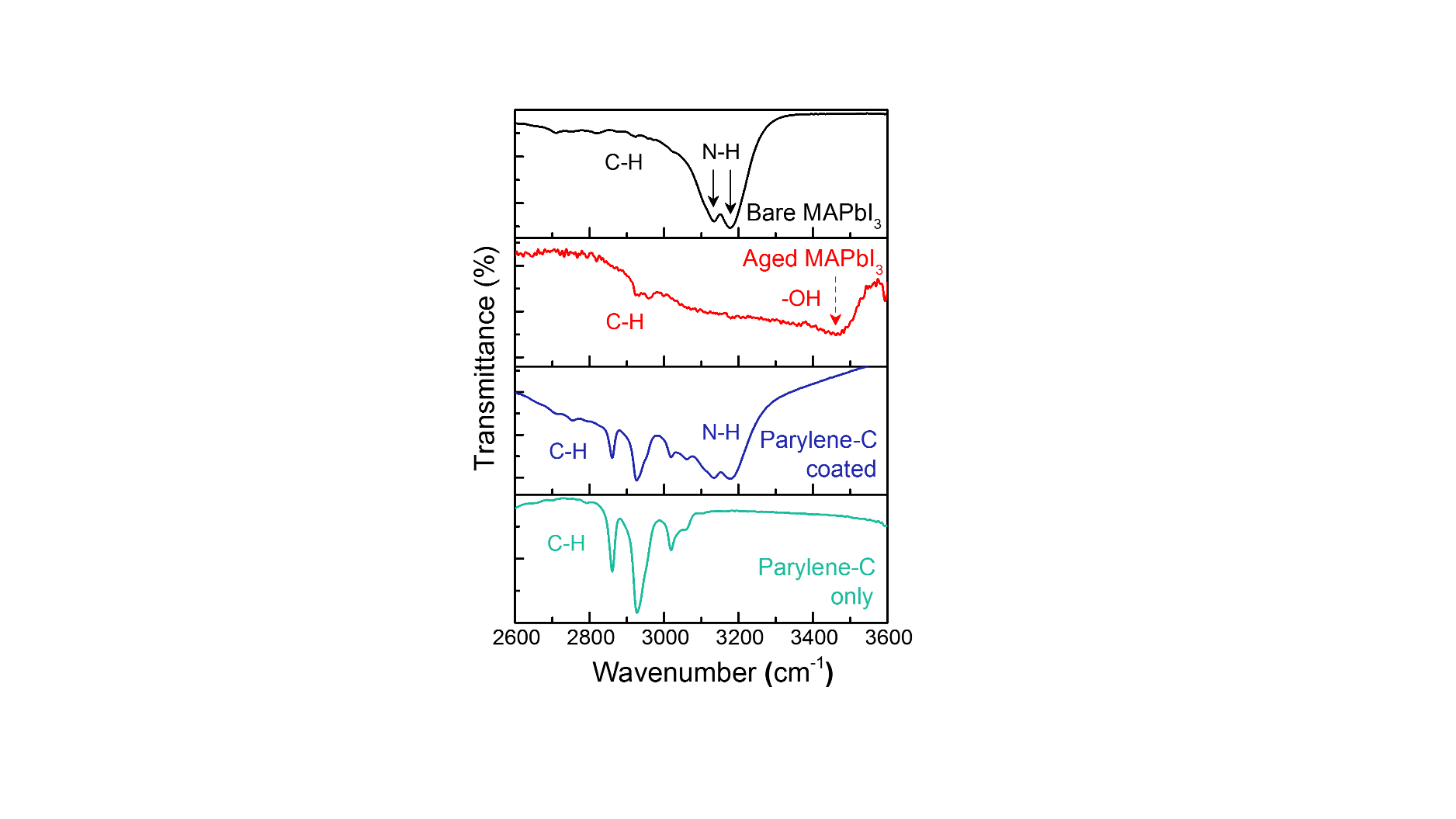
**

**Figure S7.** FTIR spectra for bare (black), aged (red), Parylene-C-coated MAPbI_3_ (blue), and Parylene-C (green).

**Reference**

[1] Lee, J., Menamparambath, M. M., Hwang, J.-Y. & Baik, S. Hierarchically Structured Hole Transport Layers of Spiro-OMeTAD and Multiwalled Carbon Nanotubes for Perovskite Solar Cells. *Chem. Sus. Chem.* **8**(14), 2358–2362 (2015).
